# Protocol for the development of a paediatric core outcome set for arthrogryposis multiplex congenita: an international multi-interest-holder consensus study

**DOI:** 10.1136/bmjopen-2026-126300

**Published:** 2026-10-15

**Authors:** Naomi Zukerman, Vasiliki Betty Darsaklis, Clarice Araujo, Sarah Cachecho, Camille Costa, Kristen Donlevie, Alicja Fąfara, Usha Kasar, Courtney Krakie, Francis Lacombe, Verity Pacey, Jaclyn Megan Sions, Noémi Dahan-Oliel

**Affiliations:** 1 Faculty of Medicine and Health Sciences, McGill University, Montreal, Quebec, Canada; 2 Clinical Research Department, Shriners Hospitals for Children Canada, Montreal, Quebec, Canada; 3 Sargent College of Health and Rehabilitation Sciences, Boston University, Boston, Massachusetts, USA; 4 Institute of Physiotherapy, Jagiellonian University Medical College, Kraków, Poland; 5 Occupational Therapy Training School & Centre, Seth GSMC & KEM Hospital, Mumbai, Maharashtra, India; 6 Department of Social Work, Shriners Hospital for Children, Sacramento, California, USA; 7 Technical Aids Services, Centre de Réadaptation Lucie-Bruneau, Montreal, Quebec, Canada; 8 Department of Health Sciences, Faculty of Medicine, Health and Human Sciences, Macquarie University, Sydney, New South Wales, Australia; 9 Department of Physical Therapy, University of Delaware, Newark, Delaware, USA

**Keywords:** Rare Diseases, REHABILITATION MEDICINE, Patient Participation

## Abstract

**Abstract:**

**Introduction:**

Ongoing research for arthrogryposis multiplex congenita (AMC) advocates for holistic and family-centred interventions. However, clinicians working with children with AMC are often met with gaps in the literature and a lack of assessment guidelines, impacting services. Current limitations in clinical practice demonstrate the need for consensus on which outcome measures to use in paediatric rehabilitation for AMC. This multiphasic study aims to develop a core outcome set (COS) for a group of rare musculoskeletal diseases (AMC), fostering standardised and inclusive care practices.

**Methods and analysis:**

This study follows a mixed-method multiphasic approach guided by the COMET Initiative’s standards for core outcome set (COS) development and COMET Handbook V.1.0. An international advisory panel was established, representing clinicians and individuals with lived experience across Canada, the United States, Poland, Australia and India. Preliminary COS concepts were derived from multidisciplinary research activities conducted between 2016 and 2024. A scoping review and advisory panel consultation supported outcome measure selection and refinement for the AMC population. Five candidate outcome measures were mapped item-by-item to the International Classification of Functioning, Disability and Health (ICF) to assess functional domain coverage and alignment with AMC rehabilitation needs. The resulting draft COS will now guide the consensus-building phase, including modified Delphi rounds, international focus groups and individual interviews, followed by external methodological appraisal.

**Ethics and dissemination:**

Ethics approval has been provided by McGill University’s Research Ethics Board (effective 21 July 2026; submission 26-05-029-01). The COS will be shared at AMC support organisation meetings, conferences and webinars, with additional knowledge mobilisation activities selected by the international advisory panel. The dissemination of findings will also be guided by the feedback provided during focus groups and interviews, to best address the needs of varied populations and ensure the accessibility of information across settings.

**OSF registration:**

10.17605/OSF.IO/QG89H.

**COMET registration:**

https://www.comet-initiative.org/Studies/Details/3378.

**STRENGTHS AND LIMITATIONS OF THIS STUDY**

- An international advisory panel, including clinicians and individuals with lived experience, is supporting the ongoing core outcome set (COS) development process to identify outcome measures relevant to arthrogryposis.
- Representation from diverse healthcare and cultural contexts across Canada, the USA, Poland, Australia and India provides global perspectives on assessment priorities and the applicability of the COS across different settings.
- Engagement of clinicians and individuals with lived experience throughout the COS development process supports the inclusion of outcomes that are meaningful to the broader arthrogryposis community.
- Purposive and snowball sampling, as well as the availability of consensus-building materials only in French and English, may influence the diversity of perspectives captured.

## Introduction

 Three hundred million people are affected by 1 of approximately 7000 rare diseases around the world.^1^ Despite this staggering statistic, the input of individuals with rare diseases is often overlooked in research.^1^ International recognition of this fact led to the United Nations’ 2021 development of the first resolution addressing the challenges of people living with a rare disease and their families.^2^ Arthrogryposis multiplex congenita (AMC) is among these rare diseases, occurring in 1 in 3000-4300 live births.^3^ AMC is a group of non-progressive musculoskeletal conditions characterised by congenital contractures in two or more different body areas.^4^

Ongoing research in AMC advocates for early and intensive rehabilitation that is family-centred.^5^ Yet, the provision of tailored family-centred care is not as straightforward as one would hope and may be limited due to experiences of exclusionary care, difficulties navigating healthcare systems and the overall sentiment of decreased agency as a caregiver.^6^ Rehabilitation practitioners working with children with rare conditions not only face the intricacies of paediatric development and family-centred care^7^ but also are equally met with gaps in the available literature and a lack of clear assessment guidelines.^8^ This may lead to challenges in identifying standardised assessments that reflect the diverse needs of each child, thereby affecting their reporting and tracking of patient progress over time. The described gaps in the literature and the lack of clear assessment guidelines highlight the need for a consensus on which outcome measures to use in paediatric rehabilitation for AMC.

Vanderhout and colleagues describe core outcome sets (COS) as ‘lists of consensus-determined outcomes to be measured and reported in all clinical research studies within a disease area’.^9^ As such, COS provide a standardised framework for identifying outcomes that are relevant to clinical practice and meaningful to patients and families, supporting treatment planning and goal setting. Their development also involves diverse interest-holders to ensure that outcomes reflect the perspectives of those affected by the condition.^10^ The COMET Initiative (ie, Core Outcome Measures in Effectiveness Trials) unites researchers, clinicians, patients and their families to facilitate the dissemination of information related to new and upcoming COS. As shown by the evolving COMET database, developing and reporting a COS is time-intensive, but possible.^11 12^ A 2022 systematic review of paediatric COS demonstrated the growing need for research in COS using adequate reporting as nearly 50% of the outcomes presented did not match COMET guidelines.^13^ As such, it is evident that it is not only the quantity of COS that can be improved but also the quality of information being shared. A significant gap remains in COS for paediatric populations with rare musculoskeletal conditions, particularly with adequate COS reporting.

Despite the recognised importance of rehabilitation in rare diseases, varied expectations and standards can lead to discrepancies between methods and measures used in practice worldwide.^3 9 14^ Meaningful involvement of people with lived experience is therefore essential to ensure that approaches to care reflect the priorities and outcomes that matter to those affected.^15^ Thus, the aim of this study is to develop a COS to be used clinically and for research purposes for children with AMC that represents the priorities of an international multi-interest-holder group comprised of individuals with AMC, caregivers and rehabilitation professionals.

## Methods and analysis

### Study design

COS development will use a mixed-methods, multiphasic design (figure 1) guided by recommendations from the COMET Initiative. The protocol was developed in accordance with the Core Outcome Set-STAndardized Protocol Items (COS-STAP) and follows the Core Outcome Set-STAndards for Development (COS-STAD)^16^ and COMET Handbook version 1.0^17^ recommendations for COS development. COS-STAD guidelines are defined as standards which represent the minimum recommendations to be considered in the process of COS development. Finally, the Core Outcome Set-STAndards for Reporting (COS-STAR) checklist will be used to ensure a complete and transparent reporting of the results for varied knowledge users.^18^ During the consensus-building stage, focus groups and individual interviews will follow the COnsolidated criteria for REporting Qualitative research (COREQ).^19^

**Figure 1:**
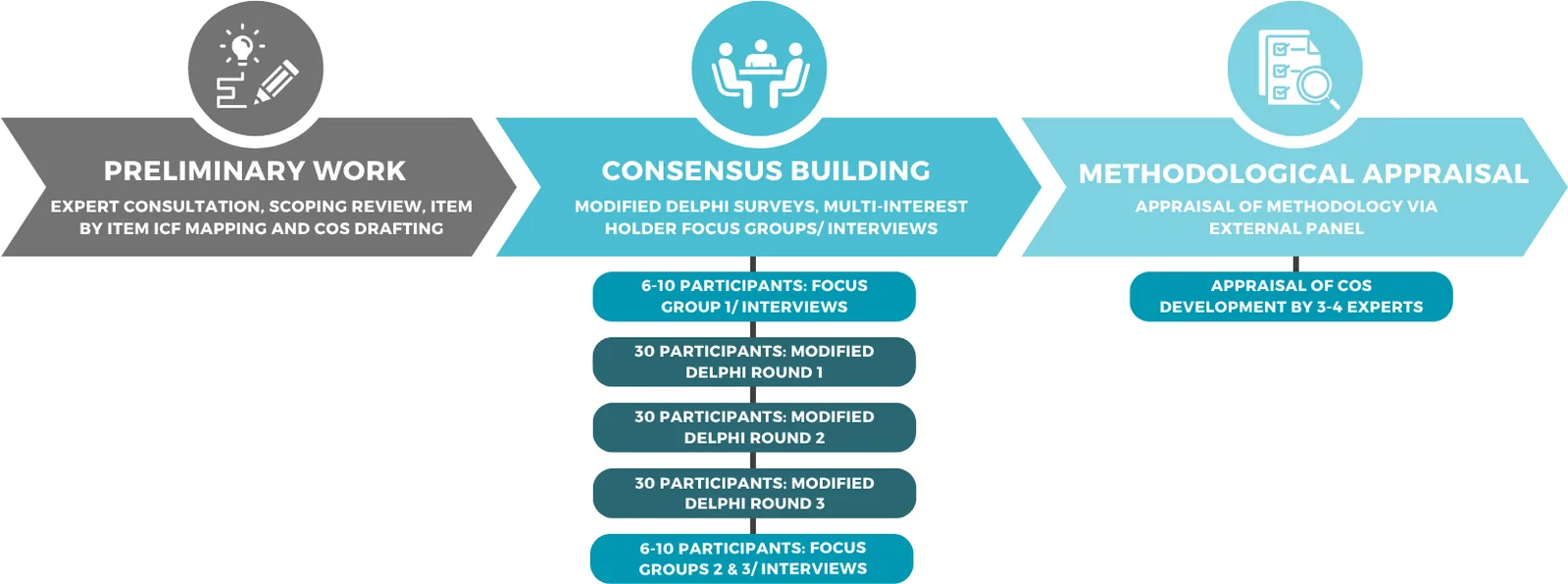
Preliminary work and current consensus-building stage involving modified Delphi surveys, international multi-interest-holder focus groups/interviews and methodological appraisal.

### Patient and public involvement statement

Patients and caregivers will be involved during the consensus-building phase through modified Delphi rounds, focus groups and individual interviews. Adults with AMC and family representatives/caregivers of children with AMC will contribute to the prioritisation and refinement of outcomes to ensure the COS reflects outcomes meaningful to the AMC community. Participants will be recruited through AMC support organisations, the international AMC consortium and collaborating networks. Infographics and other knowledge translation (KT) media selected by the participants of the focus groups will be used to share the findings of the multiphasic study. National AMC support groups, websites and social media channels (eg, Arthrogryposis Multiplex Congenita Support Inc.) will equally be consulted to disseminate the results to the widest audience.

### Study procedures

#### Preliminary work (January 2025-August 2026)

Preliminary work contributed to the identification of candidate outcomes and measures. This data directly informed the design of next steps including planned focus groups, individual interviews and online Delphi surveys, described below.

##### Data synthesis and identification of rehabilitation needs

Preliminary COS concepts were informed by a series of activities from 2016 to 2024 involving clinicians, researchers, youth with AMC and caregivers. These included a 2016 knowledge exchange day, qualitative interviews and 2017 focus groups conducted at the AMC Support Inc. annual meeting^20^ and an international clinician survey on current practice patterns.^21^ Existing AMC common data elements,^22^ including registry-recommended outcome measures, as well as the 2025 Consensus-Based Rehabilitation Recommendations,^23^ were also synthesised. Following a pooling of findings (online supplemental file 1; tables 1–4), five candidate outcome measures were identified: (1) Pediatric Outcomes Data Collection Instrument (PODCI), (2) Ages and Stages Questionnaire (ASQ-3), (3) EuroQol 5-Dimension Youth version (EQ-5D-Y), (4) Assessment of Life Habits paediatric version (LIFE-H) and (5) Patient-Reported Outcomes Measurement Information System short forms (PROMIS upper extremity function, mobility, peer relationships, fatigue and pain interference).

##### Expert consultation

An international advisory panel comprising 10 members from Australia, Canada, India, Poland and the United States was established. The panel includes clinicians and individuals with lived experience (people with AMC and caregivers) who will provide input during the planning of all study phases. The advisory panel appraised the preliminary domains and outcome measures with respect to relevance and applicability to paediatric AMC. The panel identified important gaps, including caregiver burden, fatigue and sleep. They also highlighted the need to better capture compensatory strategies, patient-reported pain and stress, patient-specific functional outcomes and more comprehensive measures of mobility. Additional recommended instruments included the Pediatric Evaluation of Disability Inventory- Computer Adaptive Test (PEDI-CAT) and pain screening tools.

##### Scoping review to identify candidate outcome measures

A scoping review was then conducted to identify candidate outcome measures for infants, children and adolescents with AMC and map the breadth and depth of available evidence. The review was structured according to Preferred Reporting Items for Systematic Reviews and Meta-Analyses guidelines with inclusion and exclusion criteria defined using the Population, Concept, Context Framework. Moreover, the review was registered on OSF (10.17605/OSF.IO/QG89H) in December 2025. A senior medical librarian developed and executed the search strategy across Medline, Embase, CINAHL, Cochrane and Global Index Medicus up to November 2025. Given the rarity of AMC, no publication date restrictions were applied, and searches were limited to the languages accessible to the research team. Study selection was conducted in duplicate at title/abstract and full-text levels, with disagreements resolved by consensus or adjudication. Data extraction was piloted, refined and completed independently by two reviewers, capturing outcomes, population characteristics and study descriptors. Extracted outcomes were standardised and consolidated through an outcome pruning process^11^ to support subsequent mapping. The results of the scoping review were shared with the advisory panel in spring 2026 to determine whether patient perspectives and clinical realities were accurately reflected. Based on their feedback, the drafted list of measures included (1) Pediatric Outcomes Data Collection Instrument (PODCI), (2) Patient-Specific Functional Scale (PSFS), (3) Patient-Reported Outcomes Measurement Information System short forms (PROMIS subsections, V.3.0 for fatigue, peer relationships, mobility, upper extremity use and pain interference), (4) Numerical Pain Rating Scale (NPRS) and (5) Ages and Stages Questionnaire (ASQ-3) to support developmental screening in younger children with AMC.

##### Mapping to the International Classification of Functioning, Disability and Health

The International Classification of Functioning, Disability and Health (ICF) classifies functioning across body functions and structures, activities, participation, environmental factors and personal factors. Selected outcome measures were mapped item by item to the ICF using updated linking rules^24^ by two trained reviewers. A total of 842 items were extracted to identify domains of coverage and gaps for the five candidate measures. Prior to full dataset mapping, a pilot sample (82/842 items), including randomly selected and hand-selected items, was manually mapped to refine coding procedures and achieve reviewer agreement. These mappings and reviewer rationale guided artificial intelligence (AI) assisted mapping of the full dataset, with iterative review and prompt refinement used to ensure alignment with coding procedures and revised ICF linking rules.^24^

##### Core outcome set drafting

Guided by this prior work, a preliminary core outcome was drafted, according to the 11 COS-STAD guidelines. Elements to consider included the practice setting for COS application, covered health condition, population, interventions, users of COS in research, representation of patients with the condition, initial list of outcomes, scoring process, inclusion/exclusion criteria for outcomes and use of accessible language.^16^ This drafted COS included the PODCI, PSFS, PROMIS subsections (V.3.0 for fatigue, peer relationships, mobility, upper extremity use and pain interference), NPRS and the ASQ-3.

### Current consensus-building stage (September 2026-December 2027)

#### Focus groups, individual interviews and modified Delphi surveys

The consensus process will begin with an initial focus group (6-10 participants) involving clinicians, caregivers and individuals with lived experience and lasting approximately 120 minutes (2 hours with resting breaks provided as needed). These discussions will be facilitated by the doctoral student, a registered occupational therapist with training in qualitative research methods and focus group facilitation, under the supervision of senior qualitative researchers. Only the research team and group participants will be present during focus groups.^19^

As part of the group introduction, demographic information will be collected from all participants to characterise the diversity of the sample (table 1). To facilitate the engagement of people with lived experience,^25^ diverse methods will be used, including Delphi surveys and focus groups (figure 1). For participants unable to attend the focus groups, an individual interview will be offered to provide equitable opportunities for participation. A bilingual interview guide will be used for focus groups/individual interviews, along with a bilingual Delphi survey system (French and English).

**Table 1:** Focus group/individual interview participants’ socio-demographic characteristics

Clinicians	Individuals with AMC	Caregivers
Geographical location	Geographical location	Geographical location
Preferred gender pronouns	Preferred gender pronouns	Preferred gender pronouns
Discipline	Age	Relationship to child
Years of experience	Type of AMC	Child’s age
Active healthcare services	Child’s type of AMC
Child’s active healthcare services

AMC, arthrogryposis multiplex congenita.

Following the focus group, up to three rounds of electronic Delphi surveys will be administered via a secure, institutionally approved platform, with each round taking approximately 15-30 minutes to complete. Approximately 30 participants are expected per survey round. Up to two post-Delphi focus groups will subsequently be conducted to (1) finalise consensus, (2) obtain open-ended qualitative feedback and (3) explore dissemination and KT strategies to best reach the wider AMC community. These final focus groups will last approximately 60-90 minutes each (1-1.5 hours), with prompts created based on the Delphi survey responses. An individual interview may be offered to participants unable to attend focus groups as an alternative to the group discussion format.

#### Sampling

Potential participants for the focus groups/individual interviews and Delphi surveys will include healthcare professionals with a minimum of 2 years of experience providing care to children with AMC. Clinicians will be recruited from multiple disciplines reflecting the multidisciplinary nature of AMC care, including occupational therapy, physical therapy, social work, physiatry and orthopaedic surgery. Participants with lived experience will include adults aged 18 years and older with AMC, as well as family representatives/caregivers (eg, parents or siblings) of children with AMC. All participants must be able to communicate in English and/or French (the languages spoken by the research team) and provide their informed consent to participate. No additional exclusion criteria are set given the rarity of AMC and the focus on ensuring that all individuals who meet the eligibility definition are represented.

For focus groups, 6-10 participants per group is recommended to support in-depth discussion while ensuring sufficient opportunity for all participants to contribute and achieve data richness.^26^ Individual interviews will be conducted as needed. For the Delphi process, sample size is not statistically determined, with Delphi studies commonly including approximately 30 participants across interest-holder groups.^27–29^ This approach ensures both depth of qualitative insight and breadth of representation appropriate for a rare condition such as AMC.

#### Recruitment

Recruitment will be conducted through passive, voluntary methods, including dissemination of a recruitment flyer via AMC support organisation websites, the international AMC consortium and the local network of the research team and collaborating institutions. The recruitment flyer will include contact information for the research team, allowing interested individuals to request additional information about the study. Eligible participants who express interest will be provided with detailed study information and invited to schedule participation in focus groups or Delphi survey activities.

#### Data collection

##### Focus groups

The initial and post-Delphi focus groups will be conducted via a secure teleconferencing platform and audio-recorded for transcription purposes. Recordings will be transcribed verbatim and analysed using inductive thematic analysis with a qualitative software, defined as an iterative process of labelling transcripts to identify recurrent patterns, concepts and meaning units across participants’ narratives. Two members of the research team will independently review and code transcripts, with discrepancies discussed until agreement is reached. Codes will be organised into a coding framework and grouped into broader themes and outcome domains. Individual interviews will be conducted in the same manner. Field notes and analytic memos will be maintained throughout data collection and analysis to document observations.

This process will directly inform the refinement of Delphi survey items by identifying gaps, redundancies and areas requiring clarification. Identified themes will be mapped onto preliminary outcome domains and used to generate, merge or revise candidate items prior to subsequent Delphi rounds.

##### Delphi survey

Participants will access the Delphi survey through a secure electronic link distributed via email. The consent form will be embedded at the beginning of the survey, and electronic consent will be required prior to proceeding to the questionnaire. The survey will be administered via a secure, institutionally approved platform, and all data will be handled in accordance with institutional confidentiality policies. Participants will rate each candidate measure using a 5-point Likert scale (Essential=5, High Priority=4, Neutral=3, Low Priority=2, Not a Priority=1), followed by a binary inclusion decision (yes/no). An a priori consensus threshold of ≥80% agreement for inclusion will be used.

Quantitative analysis will consist of descriptive statistics, including frequencies, proportions and measures of central tendency for each item. Where appropriate, subgroup analyses will be conducted (eg, clinicians, caregivers and individuals living with AMC) to explore differences in priorities across interest-holder groups. The primary outcome of the Delphi process will be the classification of each candidate measure into one of three categories: (1) consensus in (≥80% agreement for inclusion), (2) consensus out (≥80% agreement for exclusion) or (3) no consensus, which will be carried forward for reassessment in subsequent rounds. This threshold was selected because agreement levels of 70-80% are commonly used in Delphi studies and COS development to indicate strong consensus among interest-holder groups.^30^ The threshold was established before data collection to enhance methodological rigour and transparency.

After each round, aggregated, anonymised summary feedback will be provided to participants to support convergence towards consensus across iterations. Data will then be exported into Excel and analysed by the advisory panel.

### Methodological appraisal (January 2028–March 2028)

As per COS-STAD guidelines,^16^ the overall COS development will be methodologically appraised by an external expert panel. This panel will be composed of three to four experts in the areas of paediatric rehabilitation, knowledge translation (KT) and/or lived experience who were not involved in the previous phases, recruited through purposive sampling through the advisory panel’s network.

The COS-STAD guidelines are divided into 11 standards based on three domains; scope specification, interest-holders involved and consensus process.^16^ For the proposed COS, the external expert panel will be asked to rate the extent to which each of the 11 standards is met using a 1-5 scale (1=strongly disagree to 5=strongly agree). Average scores for each standard will be calculated based on the number of respondents. Adherence to guidelines and quality of the COS development will be determined as follows. Standards with an average appraisal score higher than 80% will be considered as ‘high quality’. Standards with average scores below 80% will be reviewed. Suggestions will be taken into consideration and changes will be made when applicable. As such, feedback from the external panel will lead to a final review of the included measures, to establish the first paediatric COS for AMC.

#### Data management

Data from focus groups/individual interviews and Delphi rounds will be transcribed and coded (study ID code for each participant) to protect participant confidentiality, and any coding lists will be password-protected and stored separately from other research materials. Audio-recordings and written documents from focus groups will be stored in a secure, access-restricted online data storage system. External collaborators will not have access to de-identified data at any point in the study. Disseminated data will be reported in aggregate form, and any quotations will be fully anonymised to maintain participant confidentiality. All de-identified study data will be retained for 7 years following the first publication, after which they will be securely destroyed in accordance with institutional and ethical guidelines. The coding list and other identifiable information will be retained only until study procedures are completed (ie, data collection and analysis), which is anticipated to occur approximately 1 year after study initiation.

## Ethics and dissemination

Ethics approval has been provided by McGill University’s Research Ethics Board (effective 21 July 2026; submission 26-05-029-01). Informed consent will be obtained and documented prior to participation in all focus groups, individual interviews and Delphi survey rounds. For online focus groups and individual interviews, a study team member will review the consent form, address participant questions and obtain documented verbal consent prior to discussions and audio-recording. For Delphi surveys, electronic consent will be obtained through an embedded consent form before participants can access the questionnaire.

Dissemination strategies will include traditional methods, such as open-access publications in paediatric/rehabilitation journals and conference presentations. Infographics, workshops and social media posts will be used to share findings of this project with AMC support groups and clinicians worldwide.

## Discussion

In recent years, COS have been developed by researchers to improve clinical practice, strengthen evidence basis and optimise patient care. Their use has been discussed in the context of several conditions such as cerebral palsy,^31^ osteogenesis imperfecta^12^ and juvenile idiopathic arthritis.^32^ However, rare diseases such as AMC present unique clinical and functional profiles that require condition-specific outcome measures, as consistently advocated by clinicians, families and individuals with lived experience.^33^ Building on recent advances, including the development of AMC-specific functional measures,^20^ this study represents an initial step towards the development of a paediatric COS, specifically for AMC. Engagement of diverse interest-holders, including clinicians and people with lived experience, will inform the selection of relevant and meaningful outcomes. This process will support the development of a COS that reflects the priorities of youth with AMC and their families and can guide future clinical practice and research.

## Supplementary material

10.1136/bmjopen-2026-126300 online supplemental file 1

## Data Availability

All data relevant to the study are included in the article or uploaded as supplementary information.
