# Supplementary material for: Protocol for the development of a paediatric core outcome set for arthrogryposis multiplex congenita: an international multi-interest-holder consensus study
Source: BMJ Open. 2026 Oct 15;16(10):e126300. doi: 10.1136/bmjopen-2026-126300 (PMC13691839; doi:10.1136/bmjopen-2026-126300)
Supplement: Supplementary file 1 [file bmjopen-2026-126300.reviewer_comments.pdf]

PEER REVIEW HISTORY

BMJ Open publishes all reviews undertaken for accepted manuscripts. Reviewers are asked to complete a checklist review form and are provided with free text boxes to elaborate on their assessment. These free text comments are reproduced below.

ARTICLE DETAILS

Title (Provisional)

Protocol for the Development of a Pediatric Core Outcome Set for Arthrogryposis Multiplex Congenita: An International Multi-Interest Holder Consensus Study

Authors

Zukerman, Naomi; Darsaklis, Vasiliki Betty; Araujo, Clarice; Cachecho, Sarah; Costa, Camille; Donlevie, Kristen; Fąfara, Alicja; Kasar, Usha; Krakie, Courtney; Lacombe, Francis; Pacey, Verity; Sions, Jaclyn Megan; Dahan-Oliel, Noémi

---

This article was not externally reviewed at BMJ Open. Protocols that have been independently assessed prior to submission to BMJ Open are usually fast-tracked to publication on the grounds that further substantial changes will not be possible. This independent assessment will usually be external, independent review for both a substantial grant award from a non-commercial or government funder and ethics approval.
