# Supplementary material for: Protocol for the development of a paediatric core outcome set for arthrogryposis multiplex congenita: an international multi-interest-holder consensus study
Source: BMJ Open. 2026 Oct 15;16(10):e126300. doi: 10.1136/bmjopen-2026-126300 (PMC13691839; doi:10.1136/bmjopen-2026-126300)
Supplement: Supplementary file 2 [file bmjopen-16-10-s001.docx]

**Table 1:** 31 Outcomes Measures Derived From 2016-2024 Research Activities (Literature Review, Clinician Survey, and Common Data Elements) Across 5 ICF Components

|  | **Outcome Measures** | **ICF Components (n=5)** | | | | | **Total (#/ 5)** |
| --- | --- | --- | --- | --- | --- | --- | --- |
| **#** | **Literature Review and Clinician Survey** | **Body Functions/ Structures** | **Activities** | **Participation** | **Personal Factors** | **Environmental Factors** |  |
| 1 | Range of Motion (ROM) | **✓** |  |  |  |  | 1 |
| 2 | Manual Muscle Testing (MMT) | **✓** |  |  |  |  | 1 |
| 3 | Functional Mobility Scale (FMS) | **✓** |  |  |  |  | 1 |
| 4 | Functional Independence Measure for Children (WeeFIM) | **✓** | **✓** |  |  |  | 2 |
| 5 | 6 Minute Walk Test | **✓** |  |  |  |  | 1 |
| 6 | 2 Minute Walk Test | **✓** |  |  |  |  | 1 |
| 7 | Kansas University Standing Balance Scale (KUSBS) | **✓** |  |  |  |  | 1 |
| 8 | Peabody Developmental Motor Scales (PDMS) | **✓** | **✓** |  |  |  | 2 |
| 9 | Alberta Infant Motor Scales (AIMS) | **✓** |  |  |  |  | 1 |
| 10 | Canadian Occupational Performance Measure (COPM) | **✓** | **✓** | **✓** | **✓** |  | **4** |
| 11 | Pediatric Evaluation of Disability Inventory (PEDI) | **✓** | **✓** |  |  |  | 2 |
| 12 | Pediatric Evaluation of Disability Inventory; Computer Adaptive Test (PEDI-CAT) | **✓** | **✓** | **✓** |  |  | **3** |
| 13 | Pediatric Outcomes Data Collection Instrument (PODCI) | **✓** | **✓** |  | **✓** |  | **3** |
| 14 | Assessment of Life Habits (LIFE-H) | **✓** | **✓** | **✓** | **✓** | **✓** | **5** |
| 15 | Patient-Reported Outcomes Measurement Information System (PROMIS short forms: upper extremity, mobility, peer relationships, fatigue, pain interference) | **✓** | **✓** | **✓** |  |  | **3** |
| 16 | Gait Analysis | **✓** |  |  |  |  | 1 |
| 17 | Grip Strength Testing | **✓** |  |  |  |  | 1 |
| 18 | Pinch Strength Testing | **✓** |  |  |  |  | 1 |
| 19 | Box and Blocks Test (BBT) | **✓** |  |  |  |  | 1 |
| 20 | Jebsen-Taylor Hand Function Test (JTHFT) | **✓** | **✓** |  |  |  | 2 |
| 21 | Functional Dexterity Test (FDT) | **✓** |  |  |  |  | 1 |
| 22 | Nine Hole Peg Test (9-HPT) | **✓** |  |  |  |  | 1 |
| 23 | Hawaii Early Learning Profile (HELP) Checklist | **✓** | **✓** | **✓** | **✓** | **✓** | **5** |
| 24 | Bruininks-Oseretsky Test of Motor Proficiency Second Edition (BOT-2) | **✓** |  |  |  |  | 1 |
| 25 | Kids-Assisting Hand Assessment (Kids-AHA) | **✓** | **✓** |  |  |  | 2 |
| 26 | ABIL-HAND Kids | **✓** | **✓** |  |  |  | 2 |
| 27 | Upper Extremity Functional Index (UEFI) | **✓** | **✓** | **✓** |  |  | **3** |
| 28 | Children's Hand-Use Experience Questionnaire (CHEQ) | **✓** | **✓** |  |  |  | 2 |
| **Additional Measures Included in CDE** | |  | | | | | |
| 29 | Ages and Stages Questionnaire (ASQ) | **✓** | **✓** | **✓** |  |  | **3** |
| 30 | Gillette Functional Ax Q (FAQ) | **✓** |  |  |  |  | 1 |
| 31 | EuroQoL Five Dimensions-Youth (EQ-5D-Y) | **✓** | **✓** |  | **✓** |  | **3** |

International Classification of Functioning, Disability and Health (ICF); Common Data Elements (CDE)

**Table 2:** Correspondence Between 31 Outcome Measures and Consensus-Based Rehabilitation Recommendations (CBRR)

|  | **Outcome Measures** | **Consensus-Based Rehabilitation Recommendations (n=16; Dahan-Oliel, 2025)** | | | | | | | | | | | | | | | | **Total # Addressed** |
| --- | --- | --- | --- | --- | --- | --- | --- | --- | --- | --- | --- | --- | --- | --- | --- | --- | --- | --- |
| **#** | **Literature Review and Clinician Survey** | Early intervention and motor development | | Interventions targeting muscle and joint function | | Orthotics | | Mobility + Assistive Devices | | Participation in areas of life (e.g., self-care, school, leisure, social) | | Pain management | | Well-being | | Perioperative rehabilitation | |  |
|  |  | **1** | **2** | **3** | **4** | **5** | **6** | **7** | **8** | **9** | **10** | **11** | **12** | **13** | **14** | **15** | **16** |  |
| 1 | Range of Motion (ROM) | **✓** | **✓** | **✓** | **✓** | **✓** |  |  |  |  |  |  |  |  |  |  | **✓** | 6 |
| 2 | Manual Muscle Testing (MMT) | **✓** | **✓** | **✓** | **✓** | **✓** |  |  |  |  |  |  |  |  |  |  | **✓** | 6 |
| 3 | Functional Mobility Scale (FMS) |  |  |  |  | **✓** |  | **✓** |  |  |  |  |  |  |  |  | **✓** | 3 |
| 4 | Functional Independence Measure for Children (WeeFIM) |  | **✓** | **✓** | **✓** | **✓** | **✓** | **✓** | **✓** |  |  |  |  |  |  |  | **✓** | **8** |
| 5 | 6 Minute Walk Test |  |  |  | **✓** | **✓** |  | **✓** |  |  |  |  |  |  |  |  | **✓** | 4 |
| 6 | 2 Minute Walk Test |  |  |  | **✓** | **✓** |  | **✓** |  |  |  |  |  |  |  |  | **✓** | 4 |
| 7 | Kansas University Standing Balance Scale (KUSBS) |  |  |  | **✓** | **✓** |  | **✓** |  |  |  |  |  |  |  |  | **✓** | 4 |
| 8 | Peabody Developmental Motor Scales (PDMS) |  | **✓** | **✓** |  |  |  |  | **✓** |  |  |  |  |  |  |  | **✓** | 4 |
| 9 | Alberta Infant Motor Scales (AIMS) | **✓** |  | **✓** |  |  |  |  |  |  |  |  |  |  |  |  |  | 2 |
| 10 | Canadian Occupational Performance Measure (COPM) |  |  |  |  |  |  |  | **✓** | **✓** |  |  |  |  | **✓** | **✓** |  | 4 |
| 11 | Pediatric Evaluation of Disability Inventory (PEDI) |  | **✓** | **✓** |  | **✓** |  | **✓** | **✓** | **✓** |  |  |  |  |  |  | **✓** | 7 |
| 12 | Pediatric Evaluation of Disability Inventory; Computer Adaptive Test (PEDI-CAT) |  | **✓** | **✓** |  | **✓** |  | **✓** | **✓** | **✓** |  |  |  |  |  |  | **✓** | 7 |
| 13 | Pediatric Outcomes Data Collection Instrument (PODCI) |  | **✓** | **✓** | **✓** | **✓** | **✓** | **✓** |  |  |  | **✓** | **✓** | **✓** | **✓** |  | **✓** | **11** |
| 14 | Assessment of Life Habits (LIFE-H) |  | **✓** | **✓** |  |  | **✓** | **✓** | **✓** | **✓** | **✓** |  |  |  | **✓** | **✓** | **✓** | **10** |
| 15 | Patient-Reported Outcomes Measurement Information System (PROMIS short forms: upper extremity, mobility, peer relationships, fatigue, pain interference) |  |  |  | **✓** | **✓** | **✓** | **✓** | **✓** | **✓** |  | **✓** | **✓** | **✓** |  |  | **✓** | **10** |
| 16 | Gait Analysis |  |  |  | **✓** | **✓** |  | **✓** |  |  |  |  |  |  |  |  | **✓** | 4 |
| 17 | Grip Strength Testing |  |  |  | **✓** | **✓** |  |  |  |  |  |  |  |  |  |  | **✓** | 3 |
| 18 | Pinch Strength Testing |  |  |  | **✓** | **✓** |  |  |  |  |  |  |  |  |  |  | **✓** | 3 |
| 19 | Box and Blocks Test (BBT) |  |  |  | **✓** | **✓** |  |  |  |  |  |  |  |  |  |  | **✓** | 3 |
| 20 | Jebsen-Taylor Hand Function Test (JTHFT) |  |  |  |  | **✓** | **✓** |  |  |  |  |  |  |  |  |  | **✓** | 3 |
| 21 | Functional Dexterity Test (FDT) |  |  |  |  | **✓** | **✓** |  |  |  |  |  |  |  |  |  | **✓** | 3 |
| 22 | Nine Hole Peg Test (9-HPT) |  |  |  |  |  |  |  |  |  |  |  |  |  |  |  |  | 0 |
| 23 | Hawaii Early Learning Profile (HELP) Checklist | **✓** | **✓** |  |  |  |  |  |  | **✓** | **✓** |  |  |  | **✓** | **✓** |  | 6 |
| 24 | Bruininks-Oseretsky Test of Motor Proficiency Second Edition (BOT-2) |  |  |  | **✓** | **✓** |  |  |  |  |  |  |  |  |  |  | **✓** | 3 |
| 25 | Kids-Assisting Hand Assessment (Kids-AHA) |  | **✓** | **✓** |  | **✓** | **✓** |  | **✓** |  |  |  |  |  |  |  | **✓** | 6 |
| 26 | ABIL-HAND Kids |  |  |  |  | **✓** | **✓** |  | **✓** |  |  |  |  |  |  |  | **✓** | 4 |
| 27 | Upper Extremity Functional Index (UEFI) |  |  |  |  | **✓** | **✓** |  | **✓** |  |  |  |  |  |  |  | **✓** | 4 |
| 28 | Children's Hand-Use Experience Questionnaire (CHEQ) |  |  | **✓** |  | **✓** | **✓** |  | **✓** |  |  |  |  |  |  |  | **✓** | 5 |
| **Additional Measures Included in CDE** | |  | | | | | | | | | | | | | | | | |
| 29 | Ages and Stages Questionnaire (ASQ) | **✓** | **✓** | **✓** |  |  | **✓** | **✓** | **✓** | **✓** |  |  |  |  |  |  | **✓** | **8** |
| 30 | Gillette Functional Ax Q (FAQ) |  |  |  | **✓** | **✓** |  | **✓** |  |  |  |  |  |  |  |  | **✓** | 4 |
| 31 | EuroQoL Five Dimensions-Youth (EQ-5D-Y) |  |  |  | **✓** | **✓** | **✓** | **✓** | **✓** |  |  | **✓** | **✓** | **✓** | **✓** |  | **✓** | **10** |

Consensus-Based Rehabilitation Recommendations (CBRR); Common Data Elements (CDE)

**Table 3:** Extraction of Potential Outcome Measures Based on ICF Components Addressed and Correspondence with CBRR

| **Potential Selection of Outcome Measures Based on ICF (≥3 components addressed)** | **# ICF Components Addressed (#/ 5)** | **# CBRR (#/ 16)** |
| --- | --- | --- |
| Canadian Occupational Performance Measure (COPM) | 4 | 4 |
| Pediatric Evaluation of Disability Inventory; Computer Adaptive Test (PEDI-CAT) | 3 | 7 |
| Pediatric Outcomes Data Collection Instrument (PODCI) | 3 | 11 |
| Assessment of Life Habits (LIFE-H) | 5 | 10 |
| Patient-Reported Outcomes Measurement Information System (PROMIS short forms: upper extremity, mobility, peer relationships, fatigue, pain interference) | 3 | 10 |
| Hawaii Early Learning Profile (HELP) Checklist | 5 | 6 |
| Ages and Stages Questionnaire (ASQ-3) | 3 | 8 |
| EuroQoL Five Dimensions-Youth (EQ-5D-Y) | 3 | 10 |
| Upper Extremity Functional Index (UEFI) | 3 | 4 |
| **Additional Outcome Measures Based on CBRR (≥8 recommendations corresponded)** | | |
| Functional Independence Measure for Children (WeeFIM) | 2 | 8 |

Consensus-Based Rehabilitation Recommendations (CBRR); International Classification of Functioning, Disability and Health (ICF)

**Table 4:** Selected Outcome Measures According to ICF Components Addressed and Correspondence with CBRR

| **#** | **Selected Outcome Measures** | **ICF Components Addressed (#/ 5)** | **Correspondence with Consensus-Based Rehabilitation Recommendations (#/ 16)** |
| --- | --- | --- | --- |
| **1** | Patient-Reported Outcomes Measurement Information System (PROMIS short forms: upper extremity, mobility, peer relationships, fatigue, pain interference) | 3 | 10 |
| **2** | Pediatric Outcomes Data Collection Instrument (PODCI) | 3 | 11 |
| **3** | Assessment of Life Habits (LIFE-H) | 5 | 10 |
| **4** | Ages and Stages Questionnaire (ASQ-3) | 3 | 8 |
| **5** | EuroQoL Five Dimensions-Youth (EQ-5D-Y) | 3 | 10 |
| **Mean** | | 3.4 | 9.8 |

Consensus-Based Rehabilitation Recommendations (CBRR); International Classification of Functioning, Disability and Health (ICF)
